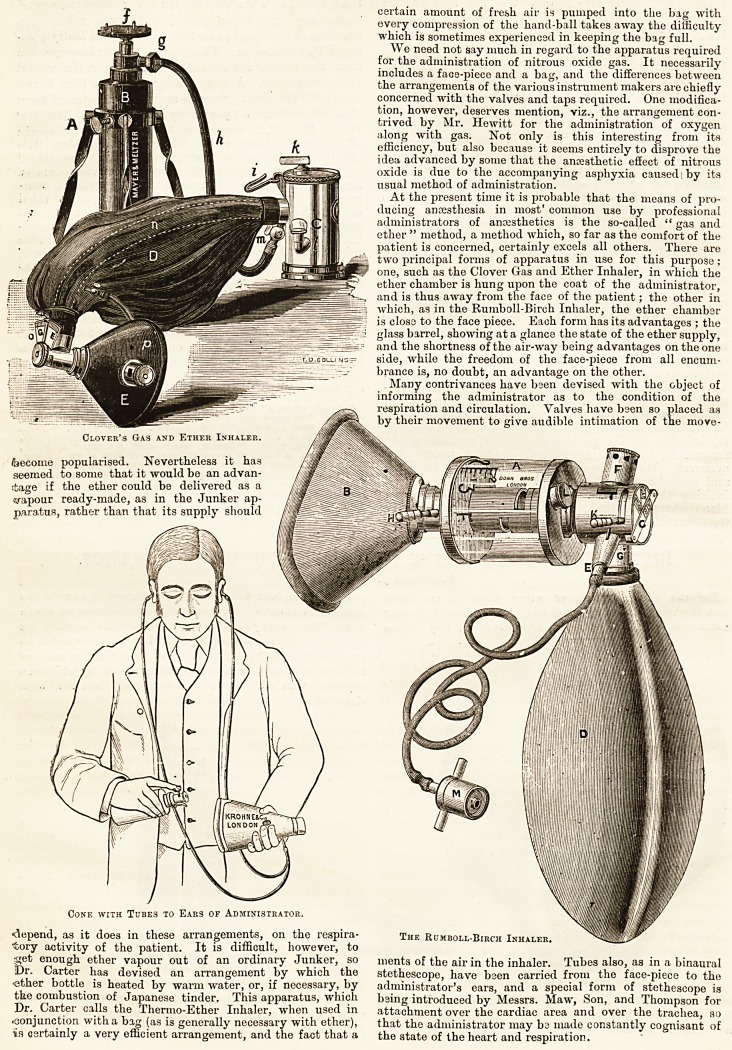# Instrumental Aids to the Administration of Anæsthetics

**Published:** 1896-10-31

**Authors:** 


					Instrumental Aids to the Administration of Anaesthetics.
{<Continued from page 66.)
For the administration of ether there is no doubt
that the most commonly used and most generally popular
instrument is the small portable Clover as produced by various
instrument makers. The proportion of ether can be very
fairly regulated, but the proportion of fresh air taken in is
not so accurately under control as might be wished, and in
prolonged administration there is an element of uncertainty
as to the amount of ether remaining in the reservoir which
is sometimes annoying. Ormsby's inhaler as modified by
Hewitt is also a very useful form, but the ether has to be
somewhat frequently renewed. Both these inhalers can now
be fitted with transparent celluloid face pieces, by which one-
objection which has been raised to their use has been
obviated.
By one or other of these inhalers the great mass ox trie
simple ether administration of the last twenty years has been
performed, and by them, especially by the Clover, ether has
sin-face was marked by ridges. The foot assumed the tliem and leaves the wire in. He never closes the wound
position of talipes valgus. Dr. F. Bauer4' reported to under any circumstances, but puts on a voluminous-
the Swedish Medical Society ten case3 of the ambulant dressing of sterilised gauze, then cotton, and lastly plas-
treatment of fractures of the lower extremity, viz., three ter of Paris bandage. The limb is afterwards suspended
fractures of both malleoli (one of them compound), one from the ceiling for several weeks without the wound.
Dupuytren's fracture, one fracture at upper and middle being inspected. A granulating wound is then found
third of tibia, one complicated fracture at middle of level with the skin. Sprains and their treatment form
tibia, one fracture of middle of tibia and upper third of the subject of a paper by Dr. J. 0. Biddle.50'
fibula, one fracture of patella, one fracture above con- If it is difficult, he says, to make a diagnosis in sprain
dyles of femur, and one case of pes valgus, where of the ankle, knee, wrist, elbow, and shoulder, it is
osteotomy was performed on the tibia and fibula. In no infinitely more so in severe sprains of the vertebral
case was it necessary to remove the plaster of Paris column. In the treatment of sprains of the joints of
bandage on account of pain or pressure. In nearly the lower extremity, he employs rest (by splints), com-
every case the patient left the bed the day after the pression (by oakum, &c.), passive motion (on the third,
bandage was put on, and then continued moving about, or fourth day), forcible manipulation, friction, and
at first with the aid of crutches, then with a cane, or massage. Dr. A. Hoffer51, of Wiirzburg, obtains excel-
with no extra support at all. The stiffness of the knee- lent results in the treatment of sprains of the ankle
joint and the atrophy of the muscles soon disappeared joint from the use of a bandage, composed of strips of
under proper treatment. To these cases Bauer has diachylon, by which the patient is enabled to walk with-
added eight others since treated successfully by the out difficulty. This form of bandage was first intro-
same method, viz., one supracondyloid fracture of the duced by Mr. E. Cotterell. If the patient is seen
femur, one V fracture of the leg, one ordinary fracture immediately after the accident, this bandage should be
of the leg, three supramalleolar fractures of tibia and applied at once ; if after the lapse of some time, and
fibula, and one Dupuytren's fracture. Dr. E. Martin48 swelling has occurred, the limb is first placed in a raised
has tried the ambulatory treatment in a limited number position, and compression employed by means of a
of suitable cases, i.e., of simple fracture of the leg seen rubber bandage, the diachylon bandage not being
early. In not a single instance has he had bad results. applied until twenty-four hours later, when the swell-
Dr. L. McLane Tiffany49 gives a description of his treat- ing has considerably diminished.
ment of compound fractures of the lower extremity , , ,, ... 10n. .. ,. T . . ., c_ 10n?
i t /n r,, ? n ? ?i 1- i /o\ 3" Annals of Surg., April. 1896, p. 44. 31 Lancet. April i5. 1896,
under lour headings: (1) Cleansing ox the limb; (4) as j 159> saoiafgow Med. Journ., April. 1896, p. 277. 33B. M. J.,
to the strangulation which will follow the swelling; April 25. 18S6, p. 1,059. 31 R. M. J., Mar. 7, 1896, p. 620. 35 ibid.,
/o\ *u ? ? i* 4_"l 1 j j i /n +!?-* n 621, 36 B. M. J., Feb. 15,1896, p. 404 37 Annals of Surg., Feb., 1896,
(3) bringing of the parts together; (4) sustaining the P 207. 36 ibid., p. 212. & Lancet. May 2, 1896, p. 1,222. 40 B. M. J.,
fragments by apparatus until definite union has April 25,1896, p. i.0E9. 41 Amals of Surg., April, 1896, p. 501. 42 Med.
occurred. He employs extensive shaving of the limb Record N. Y.. April 4,1816, p. 472. 43 Arcbiv. fur Unfallbetlkunde Bd.
i p i ? ,. i n l-i -i i I. Heft. 1. and Epit. B. M. J,, May 2, 1896 44 B. M. J., Apnl 18, 1896-,
and use ot antiseptics, and makes free the bones by p# y63. ?Archiv. fur Klin. Ohir. Bd. XLIX.. Heft.. 2, and Annals off
incising the deep fascia to prevent extravasation of Surg., Feb., 1896. p. 242. 46 Lancet, May 2, 1896, p. 1,226. 47 Amals of
blnnrl nlrrno- tliA limVi Thf> "hrnipsj tlipn primp pnsilv Surg1., Feb., 1896, p. 240. 48 Annals of Snrg., April, 1896, p. 462.
DlOOCl aio:|ig tne llmD. e? nones Lnen come easily p. 449 Tberapent. Gaz , April 15, 1896, p. *17. 61 Med.
together. If there is much deformity, however, he wires Wtek, April a, 1896, p. 167.
Instrumental Aids to the Administration of Anaesthetics.
[Continued from page 66.)
For the administration of ether there is no doubt prolonged administration there is an element of uncertainty
that the most commonly used and most generally popular as to the amount of ether remaining in the reservoir which
instrument is the small portable Clover as produced by various is sometimes annoying. Ormsby's inhaler as modified by
Hewitt is also a very useful form, but the ether has to be
somewhat frequently renewed. Botli these inhalers can now
be fitted with transparent celluloid face pieces, by which one
objection which has been raised to their use has been
obviated.
Ormsby's Inhaler Modified by Hewitt. Carter's Thermo-Inhaler.
instrument makers. The proportion of ether can be very By one or other of these inhalers the great mass ox tne
fairly regulated, but the proportion of fresh air taken in is simple ether administration of the last twenty years has been
not so accurately under control as might be wished, and in performed, and by them, especially by the Clover, ether has
82 THE HOSPITAL. Oct. 31, 1896.
/become popularised. Nevertheless it has
seemed to some that it would be an advan-
tage if the ether could be delivered as a
vapour ready-made, as in the Junker ap-
paratus, rather than that its supply should
depend, as it does in these arrangements, on the respira-
tory activity of the patient. It is difficult, however, to
get enough ether vapour out of an ordinary Junker, so
Dr. Carter has devised an arrangement by which the
<ether bottle is heated by warm water, or, if necessary, by
the combustion of Japanese tinder. This apparatus, which
Dr. Carter calls the Thermo-Ether Inhaler, when used in
?conjunction with a bag (as is generally necessary with ether),
is certainly a very efficient arrangement, and the fact that a
certain amount of fresh air is pumped into the bag with
every compression of the hand-ball takes away the difficulty
which is sometimes experienced in keeping the bag full.
We need not say much in regard to the apparatus required
for the administration of nitrous oxide gas. It necessarily
includes a face-piece and a bag, and the differences between
the arrangements of the various instrument makers are chiefly
concerned with the valves and taps required. One modifica-
tion, however, deserves mention, viz., the arrangement con-
trived by Mr. Hewitt for the administration of oxygen
along with gas. Not only is this interesting from its
efficiency, but also because it seems entirely to disprove the
idea advanced by some that the anaesthetic effect of nitrous
oxide is due to the accompanying asphyxia caused i by its
usual method of administration.
At the present time it is probable that the means of pro-
ducing anaesthesia in most' common use by professional
administrators of anaesthetics is the so-called " gas and
ether " method, a method which, so far as the comfort of the
patient is concerned, certainly excels all others. There are
two principal forms of apparatus in use for this purpose;
one, such as the Clover Gas and Ether Inhaler, in which the
ether chamber is hung upon the coat of the administrator,
and is thus away from the face of the patient; the other in
which, as in the Rumboll-Birch Inhaler, the ether chamber
is close to the face piece. Each form has its advantages ; the
glass barrel, showing at a glance the state of the ether supply,
and the shortness of the air-way being advantages on the one
side, while the freedom of the face-piece from all encum-
brance is, no doubt, an advantage on the other.
Many contrivances have bsen devised with the object of
informing the administrator as to the condition of the
respiration and circulation. Valves have been so placed as
by their movement to give audible intimation of the move-
ments of the air in the inhaler. Tubes also, as in a binaural
stethescope, have been carried from the face-piece to the
administrator's ears, and a special form of stethescope is
being introduced by Messrs. Maw, Son, and Thompson for
attachment over the cardiac area and over the trachea, so
that the administrator may be made constantly cognisant of
the state of the heart and respiration.
certain amount of fresh air is pumped into the bag with
every compression of the hand-ball takes away the difficulty
which is sometimes experienced in keeping the bag full.
We need not say much in regard to the apparatus required
for the administration of nitrous oxide gas. It necessarily
includes a face-piece and a bag, and the differences between
the arrangements of the various instrument makers are chiefly
concerned with the valves and taps required. One modifica-
tion, however, deserves mention, viz., the arrangement con-
trived by Mr. Hewitt for the administration of oxygen
along with gas. Not only is this interesting from its
efficiency, but also because it seems entirely to disprove the
idea advanced by some that the anaesthetic effect of nitrous
oxide is due to the accompanying asphyxia causedi by its
usual method of administration.
At the present time it is probable that the means of pro-
ducing anoesthesia in most' common use by professional
administrators of anaesthetics is the so-called " gas and
ether " method, a method which, so far as the comfort of the
patient is concerned, certainly excels all others. There are
two principal forms of apparatus in use for this purpose;
one, such as the Clover Gas and Ether Inhaler, in which the
ether chamber is hung upon the coat of the administrator,
and is thus away from the face of the patient; the other in
which, as in the Rumboll-Birch Inhaler, the ether chamber
is close to the face piece. Each form has its advantages ; the
glass barrel, showing at a glance the state of the ether supply,
and the shortness of the air-way being advantages on the one
side, while the freedom of the face-piece from all encum-
brance is, no doubt, an advantage on the other.
Many contrivances have been devised with the object of
informing the administrator as to the condition of the
respiration and circulation. Valves have been so placed as
by their movement to give audible intimation of the move-
Clover's Gas and Ether Inhaler.
/become popularised. Nevertheless it has
seemed to some that it would be an advan-
tage if the ether could be delivered as a
vapour ready-made, as in the Junker ap-
paratus, rather than that its supply should
Cone with Tubes to Ears of Administrator.
depend, as it does in these arrangements, on the respira- the Rumboll-Birch Inhaler.
tory activity of the patient. It is difficult, however, to
get enough ether vapour out of an ordinary Junker, so ments of the air in the inhaler. Tubes also, as in a binaural
Dr. Carter has devised an arrangement by which the stethescope, have been carried from the face-piece to the
?ether bottle is heated by warm water, or, if necessary, by administrator's ears, and a special form of stethescope is
the combustion of Japanese tinder. This apparatus, which being introduced by Messrs. Maw, Son, and Thompson for
Dr. Carter calls the Thermo-Ether Inhaler, when used in attachment over the cardiac area and over the trachea, so
?conjunction with a bag (as is generally necessary with ether), that the administrator may bs made constantly cognisant of
is certainly a very efficient arrangement, and the fact that a the state of the heart and respiration.

				

## Figures and Tables

**Figure f1:**
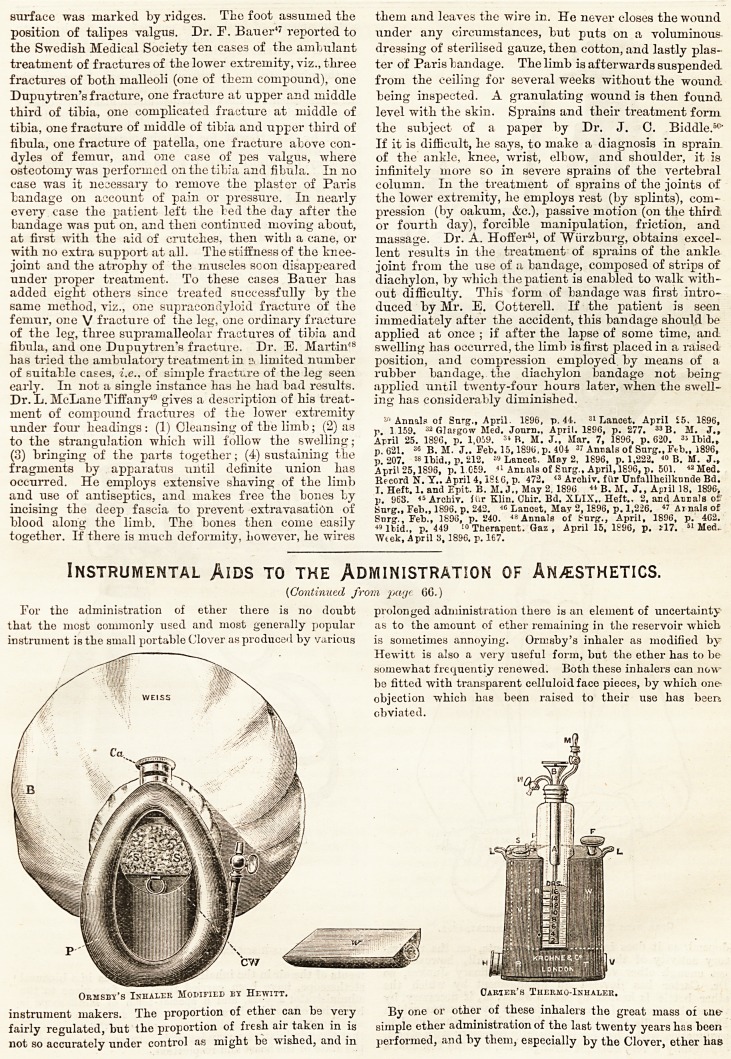


**Figure f2:**